# HSV-Encephalitis Reactivation after Cervical Spine Surgery

**DOI:** 10.1155/2019/2065716

**Published:** 2019-04-10

**Authors:** Joshua E. Heller, Geoffrey Stricsek, Lauren Thaete

**Affiliations:** ^1^Jefferson University, Department of Neurological Surgery, USA; ^2^Jefferson College of Biomedical Sciences, USA

## Abstract

**Background:**

Herpes simplex virus encephalitis (HSVE) is a viral neurological disorder that occurs when the herpes simplex virus (HSV) enters the brain. The disorder is characterized by the inflammation of the brain and a significant decline in mental status. HSVE reactivation after neurosurgery, although rare, can cause severe neurological deterioration. The high morbidity rate among untreated patients necessitates prompt diagnosis and management.

**Case Description:**

We report a case of a 78-year-old woman with no known prior history of HSVE and declining mental status eleven days after a posterior C3-T1 decompression and instrumented fusion following resection of an intradural extramedullary tumor, confirmed to be meningioma on final pathology. Reactivation of HSV-1 encephalitis was suspected to be the underlying cause of her symptoms, though MRI scans of the brain for HSVE were negative. The patient reacted positively to a 21-day treatment of acyclovir and was discharged with a neurological status comparable to her preoperative baseline. This case contributes to the literature in that it is the first reported instance of HSVE reactivation after intradural cervical spinal surgery with negative MRI findings.

**Conclusion:**

We recommend utilizing multiple tests, including PCR, EEG, and MRI, for postoperative neurosurgery patients that have decreased mental status in order to quickly and correctly diagnose/treat patients who are HSVE positive. Clinicians should consider the possibility of receiving false-negative results from PCR, CSF, EEG, or MRI tests before terminating treatment for HSVE reactivation.

## 1. Introduction

Herpes simplex virus encephalitis (HSVE) is a neurological disorder caused by the activation of the herpes simplex virus (HSV) in the brain. Few reports have been published on HSVE reactivation in patients with no known history of HSV or HSVE following cervical spinal surgery. While HSVE reactivation is rare in postoperative patients, the high morbidity rates among untreated patients necessitate a quick diagnosis [1]. We present an unusual case of a 78-year-old woman with HSVE reactivation and negative MRI scans of the brain following cervical spine surgery. We highlight the successful treatment for HVSE and recommend criteria in accordance with relevant literature for diagnosing HVSE in postoperative patients.

## 2. Case Presentation

### 2.1. History

A 78-year-old woman with an unremarkable past medical history presented to the clinic with symptoms of progressively worsening myelopathy including gait dysfunction and impairment of upper extremity fine motor skills. Noncontrast MRI of the cervical spine demonstrated multilevel degenerative disease and a dorsal intradural extramedullary lesion extending from C3-C6.

### 2.2. Operation

The patient underwent an elective posterior C3-7 decompression, C3-T1 instrumented fusion, and resection of intradural tumor. Final pathology was psammomatous meningioma. The patient tolerated the procedure well and postoperatively was transferred to the neurological ICU for close monitoring.

### 2.3. Postoperative Course

**Figure 1:**
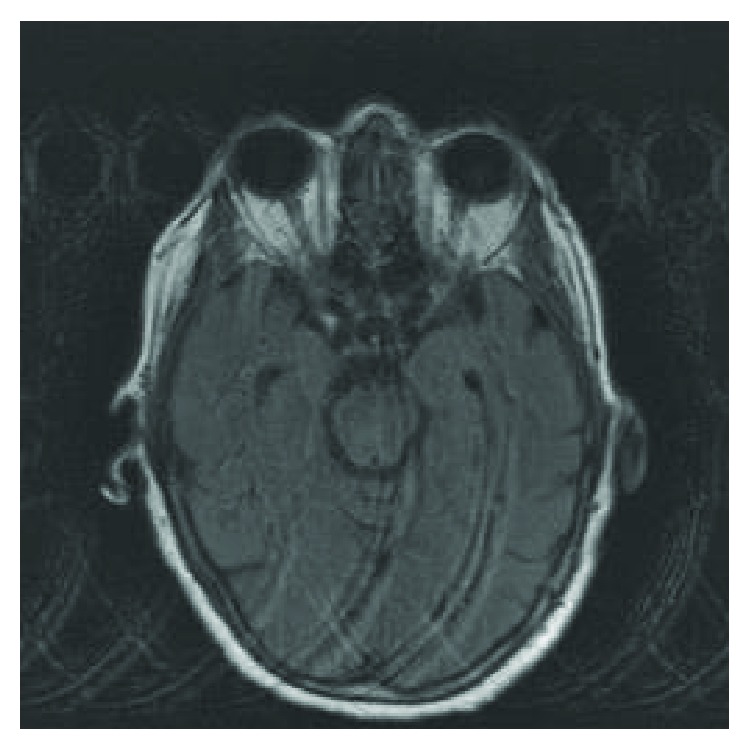
Axial T2-weighted sequence through the midbrain.

**Figure 2:**
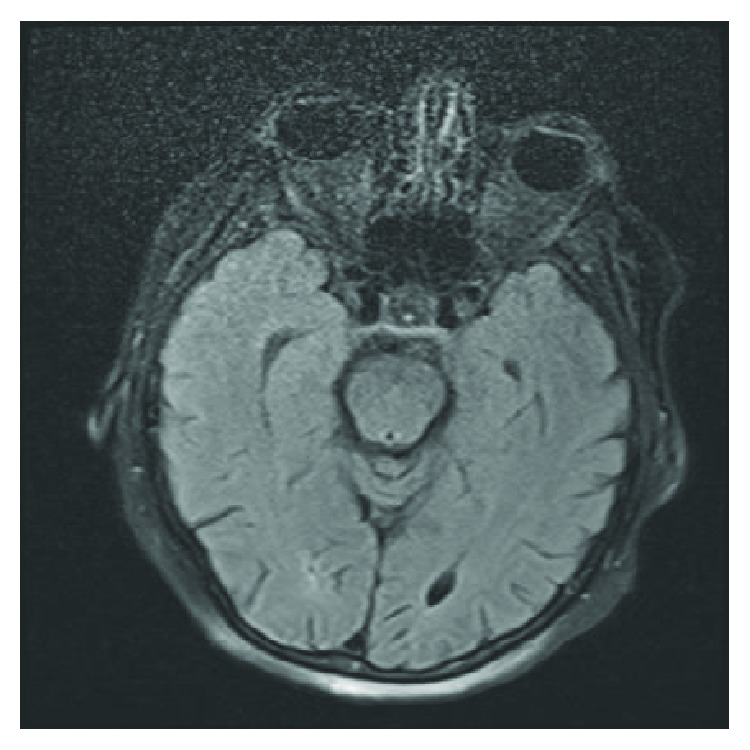
Axial T1-weighted sequence through the midbrain.

The patient was initially discharged from the hospital to an inpatient rehabilitation facility on POD 6. At the time of discharge, she was awake, oriented, and followed commands in all extremities with some mild weakness in the right deltoid and biceps, graded 4/5; the remaining muscle groups were 5/5. On POD 10, the patient developed progressive lethargy and was readmitted to the hospital for further evaluation. Upon readmission, she opened her eyes to verbal command, had incomprehensible speech, and would move all extremities spontaneously with strength 3/5 but did not follow commands. She was afebrile with WBC = 6.9 and no metabolic abnormalities. Given her recent intradural surgery, a lumbar puncture was performed. CSF cytology demonstrated 397 WBC, 20 RBC, 291 protein, and 40 glucose. CSF PCR was positive for HSV 1. Interestingly, intracranial imaging did not demonstrate the typical findings associated with herpes encephalitis (Figure 1). She was initially placed on broad spectrum antibiotics in addition to antiviral therapy. She was also connected to continuous EEG monitoring, found to be in status epilepticus, and required escalating therapy to the point of intubation with midazolam infusion. Seizure control was ultimately achieved, and she was maintained on levetiracetam 1500 mg q12H for 30 days and lacosamide 200 mg q12H for 7 days. The remainder of her infectious work-up was unremarkable, allowing her to be narrowed to only a 21-day course of IV acyclovir 500 mg q12H. Due to acute respiratory failure from encephalopathy, she underwent tracheostomy and PEG placement (POD 29); she underwent repeat MRI approximately 2 weeks after HSV diagnosis which still lacked typical findings of HSV (Figure 2). However, she continued to improve clinically; at the time of discharge, she would open her eyes spontaneously and followed simple commands in all extremities. Approximately 3 months out from being diagnosed with HSV encephalitis, she was oriented twice and followed commands in all 4 extremities with 5/5 strength in the bilateral upper and 3/5 in the bilateral lower extremities.

At a three-month follow-up, the patient presented no new symptoms. The PEG was still in place and EEG revealed excessive theta waves during wakefulness and bilateral midtemporal delta slowing (left more prevalent than right). Though the patient had dysarthria and poor attention/processing, she followed commands in all 4 extremities consistent with her last evaluation. Vimpat 200 BID and 1500 mg of Keppra were prescribed for ongoing clonus spasticity. All other medications were continued as prescribed. In the following month, the patient had a normal LOC, very slight dysarthria, and improved situational awareness. No recent seizures had occurred. Lacosamide was reduced to 150 mg every 12 hours and Vimpat was reduced to 150 BID. Upon final examination, she followed commands in all 4 extremities with 5/5 strength in the bilateral upper and 4-/5 in the bilateral lower extremities.

## 3. Discussion

Herpes simplex virus encephalitis (HSVE) is a rare viral infection of the central nervous system that can be caused by the reactivation of the HSV-1 in asymptomatic patients [2, 3]. Though HSVE affects fewer than 4 people for every 1,000,000 worldwide, the 70% mortality rate associated with untreated patients necessitates quick diagnosis [1, 4]. Though the specific mechanism in which HSV-1 infiltrates the central nervous system (CNS) is not known, it is believed to be secondary to retrograde transport of HSV-1 DNA through olfactory/trigeminal nerves or hematogenous dissemination [1, 5]. Once HSV-1 has infiltrated a neuron, the viral DNA encodes for a regulatory control protein, gC-1, which decreases the cell's control of autologous compliment system and produces symptomatic responses [3]. Most surgical procedures can aggravate immune response allowing for the reactivation of HSV-1 in trigeminal ganglia in HSVE-specific CD8+T cells [1]. Any neurosurgical intervention could lead to immunodeficient status in the early postoperative period; in spinal patients, this may occur after surgery for intradural/intramedullary tumors as well as after degenerative pathologies [2, 6, 7]. Previous cases indicate that surgery is a precipitating factor of HSV reactivation ([5]; Kim 2013).

Previous studies have recommended the use of prophylactic antiviral treatment for patients who have a known history of HSV-1 or HSVE prior to and following any surgical procedure [8]. For patients without a previous history of HSV-1 or HSVE, a significant decline in mental status (≥24h), documented fever (≥100.4°F), generalized seizures, CSF WBC (≥5 cubic mm), and an abnormality of brain parenchyma on neuroimaging (MRI/CT) or an abnormality on electroencephalography (EEG) that is consistent with encephalitis are the current diagnostic criteria for clinicians to suspect HSVE reactivation in patients [9, 10]. These diagnostic criteria, although invaluable, have been reported to produce occasional false-negative MRI, CSF, and PCR exams in postoperative neurosurgical patients [8, 11–19].

Since the diagnosis of HSVE can be challenging, advanced laboratory investigations might be required. In addition to the criteria provided above, melting point analysis of CSF or *artus* HSV-1/-2 PCR kitQ Qiagen DNA isolation procedures can detect both herpes virus-6 and varicella zoster virus [20]. Inexpensive antigen-specific immunoblotting, such as DNA immunostaining with avidin-biotin-peroxidase, can also be performed to confirm the presence of nervous system infections with positive IgG, IgM, or p53 results [21, 22]. These advanced immunostaining methods use DNA-directed immobilization of specific exons and immunoreactivity to the presence or absence of specific proteins like glial fibrillary acidic proteins (GFAP) [21, 23]. Because HSV-1 is characterized by the protein formation of the C4b2a-complex, DNA-based nano-immunoassay could become an invaluable diagnostic test for the presence of HSV-1 prior to a surgical procedure [3, 23].

Although we present a case of a 78-year-old patient, aspects of prognosis and morbidity may differ between populations of patients [5, 20]. The literature suggests a tendency to affect “frail subjects, with peaks in the elderly and pediatric populations [5, 24, 25]”; hence, a high index of suspicion should be considered for those at high risk. A systematic review of the literature on patients undergoing surgery for degenerative cervical myelopathy suggests that though duration and severity of symptoms related to neurological procedures are more closely associated with surgical outcomes, elderly patients and positive smoking status may have predictive value for comorbidities following surgery [7]. Early detection of pathological patterns in neonatal patients and pediatric patients can be challenging due to the wide range of etiologies that could be linked to HSVE and the limitations, mainly cost and safety, of using MRI on young patients (Bourgeois 1999; [17, 26, 27]). Even with consideration for pediatric patients with known history of HSV-1, pre-, peri-, and postoperative treatment for HSV following neurosurgery still poses a high risk for developing severe postoperative clinical deterioration (Bourgeois 1999; [5, 27]). The outcomes of elderly patients also could present a higher possibility of comorbidities; however, several cases report complete recovery from HSVE following neurosurgery ([7]; Pena 1987; Koskiniemi 1996). Though age may affect the diagnostic tests used and recovery, the accuracy and speed of treatment following initial symptoms are the best predictors of clinical outcomes [5, 7, 25]. Our case is a unique contribution to the literature because it demonstrates a presumed case of HSV and resultant herpes encephalitis with no significant MRI findings in the setting of recent intradural cervical spinal surgery, an event not previously reported in the literature.

## 4. Conclusion

Our case outlines the postoperative course, diagnosis, and treatment of a patient with no known history of HSVE who developed neurological abnormalities following a recent intradural cervical spinal surgery with negative test results. Though the reactivation of HSVE in patients after cervical surgery is rare, the ability to quickly recognize and diagnose HSVE in postoperative patients is crucial when considering the mortality rate of untreated patients with HSVE. For postoperative symptomatic patients, PCR, CSF, EEG, and MRI testing should be performed to determine the presence of HSVE. Because false-negative findings for HSVE can occur in each of these tests, it is recommended that additional tests be repeated in the absence of an alternative diagnosis for decline in mental status. Clinicians should also be cautious of prematurely terminating treatment for HSVE due to false-negative exams. As demonstrated in our case, it is important that clinicians be quick to identify and diagnose HSVE in the setting of patients with no known history of HSVE and HSV-1.
